# Portal vein thrombosis; risk factors, clinical presentation and treatment

**DOI:** 10.1186/1471-230X-7-34

**Published:** 2007-08-15

**Authors:** Kirstine K Sogaard, Lone B Astrup, Hendrik Vilstrup, Henning Gronbaek

**Affiliations:** 1 Department of Medicine V (Hepatology and Gastroenterology), Aarhus University Hospital, 8000 Aarhus C, Denmark

## Abstract

**Background:**

Portal vein thrombosis (PVT) is increasingly frequently being diagnosed, but systematic descriptions of the natural history and clinical handling of the condition are sparse. The aim of this retrospective study was to describe risk factors, clinical presentation, complications and treatment of portal vein thrombosis in a single-centre.

**Methods:**

Sixty-seven patients were identified in the electronic records from 1992 to 2005. All data were obtained from the patient records.

**Results:**

One or more risk factors (e.g. prothrombotic disorder or abdominal inflammation) were present in 87%. Symptoms were abdominalia, splenomegaly, fever, ascites, haematemesis, and weight loss. Abdominalia and fever occurred more frequently in patients with acute PVT. Frequent complications were splenomegaly, oesophageal- and gastric varices with or without bleeding, portal hypertensive gastropathy and ascites. Varices and bleeding were more frequent in patients with chronic PVT. Patients who received anticoagulant therapy more frequently achieved partial/complete recanalization. Patients with varices who were treated endoscopically in combination with β-blockade had regression of the varices. The overall mortality was 13% in one year, and was dependent on underlying causes.

**Conclusion:**

Most patients had a combination of local and systemic risk factors for PVT. We observed that partial/complete recanalization was more frequent in patients treated with anticoagulation therapy, and that regression of varices was more pronounced in patients who where treated with active endoscopy combined with pharmacological treatment.

## Background

Over the last years, portal vein thrombosis (PVT) is increasingly frequently being diagnosed by wide use of ultrasound-Doppler equipment. Recently, the lifetime risk of getting PVT in the general population is reported to be 1% [1]. The condition, thus, attracts more focus. The conception of the severity of the problem among treating physicians varies between desolate and unimportant. The recommended therapeutic approach varies between one of expectancy and active intervention aimed at supposed risk factors and complications. Accordingly, there is a need for a basis for clinical decisions.

Risk factors and complications to PVT have become better defined although most of the information derives from patients with cirrhosis [2,3]. Still, the rational management of PVT is still not adequately understood, and there have been no controlled studies. Based on some case-series, anticoagulation may be associated with recanalization of both acute [4,5] and chronic thrombosis [6]. Band ligation remains central in treating acute variceal bleeding. Secondary prophylaxis of rebleeding with ligation is effective; however, there are insufficient data on β-blockade alone or in combination with endoscopy [7-9].

PVT seems most often to develop in the presence of both systemic and local risk factors [10,11], but the relative importance of these factors for the course and treatment strategy of the condition remains unclear.

Thus, there are several unresolved issues regarding PVT that due to the rarity of the condition cannot be clarified by large trials.

Therefore, the aim of this case-series was to describe risk factors, clinical presentation, complications, treatment and outcome in patients with PVT in a tertiary university hospital.

## Methods

Sixty-seven patients were identified in the computerised hospital administrative registration system by the ICD10 classification code I81.9. All cases from January 1992 to December 2005 of extrahepatic portal vein thrombosis or intrahepatic portal vein thrombosis were included. All registered diagnoses were based on either ultrasound with Doppler, CT-angiography or MRI. Diagnostic inclusion criteria were partial or complete thrombosis with the extension of the thrombus defined. Follow-up elapsed from the time of admission, and lasted to either death or December 31^st ^2005.

The following data were extracted from the clinical records: sex, age, risk factors (prothrombotic tendency, cirrhosis, cancer, abdominal inflammation, infection, surgical abdominal intervention, none), clinical presentation; abdominalia (= abdominal pain, loss of appetite, nausea, vomiting or diarrhoea), splenomegaly, fever, ascites (tense/soft, diagnosed by clinical evaluation or ultrasound), haemorrhage (= haematemesis, melaena or rectal bleeding), presentation of PVT (acute < 60 days or chronic > 60 days [4]), complications (oesophageal- and gastric varices, variceal haemorrhage, portal hypertensive gastropathy, ascites), extension of the thrombus (intra- and/or extrahepatic, superior mesenteric vein or splenic vein), information on coagulation disorders, imaging methods (Doppler ultrasound, CT angiography, MRI, and endoscopy), treatment (anticoagulation, thrombolysis, sclerotherapy, banding, β-blockade, long-acting nitrates and transjugular intrahepatic portosystemic shunting (TIPS)), the course of varices, recanalization and finally the cause of death.

Diagnosis of portal hypertensive gastropathy and grading of oesophageal- and gastric varices was made by means of endoscopy. Thrombophilia investigation was performed by using citrated plasma and heparin-flouride containing plasma collected by venipuncture and kept at -80°C until analysis. The protocol for sample collection and processing, as well as data interpretation, has been reported in previous publications [12-14].

Thrombophilia testing included collection for antithrombin (AT), homocystein, protein C, free protein S, factor V (FV) Leiden, prothrombin G20210A, Von Willebrand-factor, activated protein C resistance, β-2 glycoprotein-1 (APA), phospholipid antibody, and lupus anticoagulant (LA). The spontaneous fibrinolytic capacity was determined by a routine fibrin plate technique.

Due to the severity of cancer and cirrhosis, we stratified the patients in two groups: 1. Patients without cancer or cirrhosis and 2. Patients with cancer or cirrhosis. Data from the two groups were analysed separately. Results for the group of patients *without *cancer and cirrhosis (n = 48) will be presented and compared with results for the group of patients *with *cancer or cirrhosis (n = 19).

All analyses were done using Stata version 9.2. Significance was calculated with Student's t-test or Chi squared test. P < 0.05 was considered statistically significant in a two-sided test.

### Ethical consideration

The Central Denmark Region Committee on Biomedical Research Ethics confirmed that no approval was required for this study.

## Results

### Basic data

For patients without cancer and cirrhosis, mean age at time of admission was 44 ± 17 SD (range 15–74). The group included 23 women and 25 men. Seventeen had acute PVT and 31 chronic PVT. There was no difference in age between genders, but higher age in patients with acute (51 ± 16) compared to chronic (40 ± 16) PVT at time of diagnosis. Mean time elapsed from time of admission was 39 ± 41 months (range 0–158).

For patients with cancer and cirrhosis, mean age at time of admission was 57 ± 12 SD (range 34–78). Six were women and 13 men. Ten had acute PVT and 9 chronic PVT. There was no difference in age between genders, or between acute and chronic PVT at time of diagnosis. Mean time elapsed from time of admission was 26 ± 27 months (range 0–92).

### Risk factors (Table 1)

**Table 1:** Risk factors of portal vein thrombosis in all patients (n = 67)

**Primary risk factor**	**All risk factors**
**Prothrombotic disorder n = 19 (28%)**	**Prothrombotic disorder n = 36 (54%)** Hyperhomocysteinemia (n = 13), antiphospholipid syndrome (n = 9), hormone replacement therapy (n = 2), Factor V Leiden mutation (n = 3), Protein C deficiency (n = 2), Polycythaemia vera (n = 2), myeloproliferative syndrome (n = 1), Protein S deficiency (n = 1), antitrombin III deficiency (n = 1), disseminated coagulation (n = 1), essential thrombocytosis (n = 1)

**Abdominal inflammation n = 13 (19%)**	**Abdominal inflammation n = 23 (34%)** Chronic pancreatitis (n = 11), Cholangitis (n = 5), acute pancreatitis (n = 2), liver abscesses (n = 2), umbilical vein catheterization (n = 1), cholecystolithiasis (n = 1), polycystic liver disease (n = 1)

**Cirrhosis n = 9 (13%)**	**Cirrhosis n = 12 (18%)**

**Cancer n = 7 (11%)**	**Cancer n = 9 (13%)** Neuro-endocrine Tumor (n = 4)*, hepatocellular carcinoma (n = 2), pancreatic cancer (n = 1), unknown primary tumour (n = 1), angiomyxoma (n = 1)

**Abdominal intervention n = 5 (8%)**	**Abdominal intervention n = 8 (12%)** Splenectomy (n = 3), cholecystectomy (n = 2), Billroth II (n = 1), radiofrequency ablation (n = 1), gastropancreaticcystotomy (n = 1)

**Abdominal infection n = 5 (8%)**	**Abdominal infection n = 9 (13%)** Bacteraemia (n = 4), portal vein phlebitis (n = 2), intestinal tuberculosis (n = 1), sepsis (n = 1), tuberculosis in psoas abscess (n = 1)

**Idiopathic n = 9 (13%)**	**Idiopathic n = 9 (13%)**

* A relative large proportion reflecting the centre's specialist function for this disease. Patients can have more than one risk factor.

Risk factors were established in 58 cases (87%). Twenty-nine cases (43%) had two risk factors, and 14 (21%) had three risk factors.

When including all risk factors, 43 cases (64%) had a local risk factor, and 28 cases (42%) had a systemic risk factor. Analyses of patients without cancer or cirrhosis showed a difference of risk factors: 27 cases (56%) had a local risk factor, and 24 cases (50%) a systemic risk factor.

### Imaging methods

In 51 (76%) patients, diagnosis was established by means of Doppler ultrasound. Of 12 patients with a negative ultrasound, 11 were diagnosed by CT angiography and one by MRI-scanning. In four patients, the diagnosis was initially established with CT or MRI. In all patients diagnosed by means of Doppler ultrasound, CT angiography was later performed. MRI-scanning was only performed in five of the patients with a positive Doppler ultrasound, and supported the diagnosis in all five cases. Sensitivity was calculated to be 81% (51/63) for Doppler ultrasound and 94% (51/54) for CT. Anatomical location was in 65 cases (97%) extrahepatic, and in two cases (3%) intrahepatic. In addition, patients with extrahepatic PVT also had intrahepatic thrombosis (54%) and/or in the splenic vein (42%), and/or in superior mesenteric vein (31%), and one patient also had thrombosis of the inferior mesenteric vein.

### Clinical manifestation (Table 2)

**Table 2:** Clinical presentation in patients with cancer or cirrhosis, and patients without cancer and cirrhosis

**Analysis**	**Patients with cancer or cirrhosis N = 19**			**Patients without cancer and cirrhosis N = 48**		
	**Presentation**			**Presentation**		

	**Acute**	**Chronic**	**N (% of total)**	**Acute**	**Chronic**	**N (% of total)**

**Abdominalia**	7 (70%)	5 (56%)	46 (63%)	16 (94%)	18 (58%)	34 (71%)
**Splenomegaly**	7 (70%)	5 (56%)	12 (63%)	10 (59%)	26 (84%)	36 (75%)
**Fever**	5 (50%)	2 (22%)	7 (37%)	10 (59%)	5 (16%)	15 (31%)
**Haemorrhage**	6 (22%)	5 (35%)	11 (58%)	0	9 (29%)	9 (19%)
**Ascites**	4 (40%)	2 (22%)	6 (32%)	8 (47%)	10 (38%)	18 (38%)
**Weight loss**	1 (10%)	2 (22%)	3 (16%)	9 (53%)	7 (23%)	16 (33%)
**Total**	10 (53%)	9 (47%)	19 (100%)	17 (35%)	31 (65%)	48 (100%)

*Data are presented as N of total number and % within the acute/chronic presentation.

In the analysis of patients without cancer or cirrhosis, 71% presented with abdominalia, 75% splenomegaly, 31% fever, 38% ascites, 19% haemorrhage and 33% weight loss. Abdominalia and fever were more frequent symptoms in the acute disease.

In the analysis of patients with cancer or cirrhosis, 63% had abdominalia, 63% splenomegaly, 37% fever, 32% ascites, 58% haemorrhage and 16% weight loss.

Patients with ascites had light to moderate ascites and 28% were treated with paracentesis.

### Complications (Table 3)

**Table 3:** Complications in patients with cancer or cirrhosis, and patients without cancer and cirrhosis

**Analysis**	**Patients with cancer or cirrhosis N = 19**			**Patients without cancer and cirrhosis N = 48**		
	**Presentation**			**Presentation**		

	**Acute**	**Chronic**	**N (% of total)**	**Acute**	**Chronic**	**N (% of total)**

**Portal hypertensive gastropathy**	4 (40%)	3 (33%)	7 (37%)	5 (29%)	16 (52%)	21 (44%)
**Gastric varices**	4 (40%)	4 (44%)	8 (42%)	4 (24%)	16 (52%)	20 (42%)
**Oesophageal varices, small**	4 (40%)	6 (67%)	10 (53%)	7 (41%)	18 (58%)	25 (52%)
**Oesophageal varices, large**	1 (10%)	1 (11%)	2 (11%)	1 (6%)	10 (32%)	1 (23%)
**Haemorrhages (1–2)**	2 (20%)	4 (44%)	6 (32%)	0	10 (32%)	10 (21%)
**Haemorrhages (repeated)**	3 (30%)	1 (11%)	4 (21%)	0	4 (13%)	4 (8%)
**Ascites**	4 (40%)	4 (44%)	8 (42%)	8 (47%)	12 (39%)	20 (42%)
**Total**	10 (53%)	9 (47%)	19 (100%)	17 (35%)	31 (65%)	51 (100%)

*Data are presented as N of total number and % within acute/chronic presentation.

Fifty-nine patients had one or more complications. Among patients without cancer or cirrhosis, 72% had oesophageal varices, 42% gastric varices, 44% portal hypertensive gastropathy, 29% variceal haemorrhage, and 42% ascites. Of 36 patients with oesophageal varices, 20 (56%) also had gastric varices. Oesophageal varices and bleeding were more frequent in patients with chronic PVT.

Among patients with cancer or cirrhosis, 63% had oesophageal varices, 42% gastric varices, 37% portal hypertensive gastropathy, 43% variceal bleeding, and 42% ascites. Of 12 patients with oesophageal varices, six also had gastric varices.

### Treatment

Twenty-seven (56%) of the patients without cancer or cirrhosis received anticoagulation therapy (vitamin K-antagonist, low molecular weight heparin or heparin), compared with only 32% of the patients with cancer or cirrhosis. One patient with extensive thrombosis received recombinant tissue plasminogen activator, and in three patients, transjugular (or transhepatic) intrahepatic portosystemic shunt was established.

Forty-five percent (10 acute and five chronic) of the patients without cancer or cirrhosis who received anticoagulation therapy (16 acute and 11 chronic) improved their portal flow with partial or complete recanalization during follow up. All four patients with cancer or cirrhosis and acute PVT (67%) who received anticoagulation therapy had partial or complete recanalization. Among such patients who did not receive anticoagulation therapy (n = 34), only 15% improved their portal flow and had any degree of spontaneous resolution of the thrombosis.

Of patients with oesophageal varices (n = 48), 60% of the patients without, and (n = 12) 75% of patients with cancer of cirrhosis were treated endoscopically with band ligation (VBL) and/or sclerotherapy, in 24% as primary prophylaxis. Forty-two percent of patients without and 17% of patients with cancer or cirrhosis were treated with β-blockers as primary prophylaxis. Of patients with one or more variceal bleeding episodes (n = 24), 92% of patients without and 80% with cancer or cirrhosis were treated with VBL or sclerotherapy, and 88% with and 90% without cancer or cirrhosis with β-blockers (24% of these also with long-acting nitrates) as secondary prophylaxis.

Of the patients with varices who were treated endoscopically, 90% of patients with and 100% without cancer or cirrhosis also received medical treatment with β-blockers, and of these, respectively 58% and 63% showed regression of varices during follow-up. Seven patients with varices received no treatment, and their varices remained unchanged or progressed.

### Mortality

Of patients without cancer or cirrhosis, eight (17%) died during follow up. The causes of death were variceal bleeding (n = 1), profuse intrahepatic bleeding after trombolysis (n = 1), infection (n = 1), pulmonary embolism (n = 1), acute myocardial infarction (n = 1), florid pseudomembraneous colitis (n = 1) and unknown (n = 2). Of patients with cancer and cirrhosis, six (32%) patients died during follow-up; causes of death were renal insufficiency (n = 2), liver failure (n = 2), cancer (1) and unknown (1).

Risk factors of PVT were cirrhosis (n = 4), prothrombotic disorder (n = 4), cancer (n = 2), unknown (n = 2), splenectomy (n = 1) and chronic pancreatitis (n = 1). Within the first year after diagnosis of PVT (and admission to our hospital), four (8%) of the patients without cancer or cirrhosis died, and five (26%) of the patients with cancer or cirrhosis died.

## Discussion

Retrospectively, we report risk factors and prognostic data on 67 patients with PVT referred to a single centre university hospital in Denmark between 1992 and 2005.

The main limitation of this study is lack of generalizability due to its retrospective nature; however, large-scale randomised trials of such rare conditions can not be conducted, and, to our knowledge, this is one of the larger retrospective studies of PVT. In accordance with previously published studies, we found that PVT developed because of several risk factors [9,15,16]. In the restricted analysis, we found a combination of local (56%) and systemic risk factors (50%). Denninger et al. [17] reported a higher incidence of systemic factors (72%).

Only 11% of our PVT patients with cirrhosis and none of those with cancer were screened for coagulation disorders. However, it is important systematically to screen patients with a life expectancy of more than 3–6 months and where anticoagulation therapy is considered relevant, and particularly cirrhosis patients, since 70% of these reportedly have a genetic thrombogenic predisposition [18].

The typical presentation of acute PVT was abdominalia, splenomegaly, fever and ascites, while the presentation of chronic PVT was abdominalia and splenomegaly together with gastrointestinal haemorrhages and ascites. We found a higher frequency of abdominalia and fever in patients with acute PVT than in those with chronic PVT. Other studies report the same presentation, though ascites is described only in few cases of acute PVT [9,15]. The high presenting occurrence of ascites among our patients may be due to the fact that we registered ascites both when detected by physical examination and by ultra sound, so that cases of slight ascites were included. The ascites was in no case tense (although some were treated with paracentesis to speed up recovery). Ascites in such patients is most likely caused by intestinal venous congestion, whereas the mechanisms leading to massive fluid retention are not activated as at sinusoidal portal hypertension.

Frequent complications during follow-up were oesophageal- and gastric varices, portal hypertensive gastropathy, bleeding from varices and ascites. A larger part of patients with chronic PVT developed oesophageal varices in comparison with patients with acute PVT. Thus, the development of varices is a time dependent phenomenon, and it is advisable to screen all PVT patients endoscopically. Twenty-nine percent of patients without cancer or cirrhosis experienced variceal bleeding (in patients with cancer or cirrhosis 53%), in only one patient fatal, in accordance with other reports [6,19].

Spontaneous resolution of the thrombosis did happen in some cases, but the frequency of partial/complete recanalization seemed to be higher in patients treated with anticoagulation therapy. The effect was seen in patients with both acute and chronic PVT. The aim of anticoagulation therapy is both to prevent further thrombosis, and potentially lead to recanalization, thereby preventing the development of portal hypertension and its complications. It has been suggested that interventional recanalization should be performed whenever the result of anticoagulation is unsatisfactory, and furthermore, that portal stents should be implanted in patients with cirrhosis [20]. One study showed no increased rate of bleeding episodes in patients with established PVT who received anticoagulant therapy [6]. However, to our knowledge, there is yet no consensus on the indication for anticoagulant therapy [9]. For primary prophylaxis, β-blockade is standard for cirrhotic portal hypertension, but the effect is not documented in PVT, and VBL may be preferable. In the acute management of variceal bleeding, vasoactive substances, antibiotics, and VBL remain central. For secondary prophylaxis of re-bleeding we used combined VBL and β-blockade ± long-acting nitrates [7,8,21,22] although evidence to support β-blockers is sparse [9]. In any case, our patients showed regression of their varices when given this combination preventive treatment.

In accordance with others, we found that the outcome of PVT in general is good, and that mortality primarily was associated to underlying cause and less to the consequences of portal hypertension [6,20,23,24]. Mortality in one year was highest in patients with cancer or cirrhosis, 26% compared with only 8% in patients without these diseases.

## Conclusion

In conclusion, most patients had a combination of local and systemic risk factors for PVT. We observed that partial/complete recanalization was more frequent in patients treated with anticoagulation therapy, and that regression of varices was more pronounced in patients who were treated with active endoscopy combined with pharmacological treatment.

## Competing interests

The authors declare that they have no competing interests.

## Authors' contributions

KKS collected data, constructed the data set, performed the analysis and drafted the manuscript. LBA, HV and HG FG conceived of the study, participated in its design and coordination and helped to draft the manuscript. All authors read and approved the final manuscript.

## Pre-publication history

The pre-publication history for this paper can be accessed here:


